# Ferroptosis Meets Cell–Cell Contacts

**DOI:** 10.3390/cells10092462

**Published:** 2021-09-17

**Authors:** Cornelia Dietrich, Thomas G. Hofmann

**Affiliations:** Institute of Toxicology, University Medical Center of the Johannes Gutenberg-University, Obere Zahlbacher Str. 67, 55131 Mainz, Germany

**Keywords:** ferroptosis, cell–cell contacts, epithelial–mesenchymal transition, cancer therapy

## Abstract

Ferroptosis is a regulated form of cell death characterized by iron dependency and increased lipid peroxidation. Initially assumed to be selectively induced in tumour cells, there is increasing evidence that ferroptosis plays an important role in pathophysiology and numerous cell types and tissues. Deregulated ferroptosis has been linked to human diseases, such as neurodegenerative diseases, cardiovascular disorders, and cancer. Along these lines, ferroptosis is a promising pathway to overcoming therapy resistance of cancer cells. It is therefore of utmost importance to understand the cellular signalling pathways and the molecular mechanisms underlying ferroptosis regulation, including context-specific effects mediated by the neighbouring cells through cell–cell contacts. Here, we give an overview on the molecular events and machinery linked to ferroptosis induction and commitment. We further summarize and discuss current knowledge about the role of cell–cell contacts, which differ in ferroptosis regulation between normal somatic cells and cancer cells. We present emerging concepts on the underlying mechanisms, address open questions, and discuss the possible impact of cell–cell contacts on exploiting ferroptosis in cancer therapy.

## 1. Introduction

Cell death can be executed in a regulated or accidental manner. Accidental cell death generally occurs after harsh conditions and proceeds uncontrolled. In contrast, regulated cell death (RCD) is executed by defined cellular programmes and—according to its nature—can be regulated by pharmacological or genetic interventions. When RCD occurs in physiological contexts, e.g., embryonic development or maintenance of tissue homeostasis, it is referred to as programmed cell death [1]. It has long been considered that apoptosis is the sole example of programmed and regulated cell death. Today we know that a myriad of different regulated cell death modalities exist, including necroptosis, parthanatos, mitochondrial permeability transition (MPT)-mediated necrosis, or ferroptosis, to name a few [2]. Each cell death pathway seems to be unique in its key elements and execution processes, although also mixed forms and crosstalk between these forms may exist. In particular, necroptosis, a well-studied cell death mechanism, as well as ferroptosis have attracted broad attention in the scientific community due to their pathophysiological roles in various human diseases, such as cardiovascular disorders and neuro-degenerative diseases [3]. However, the therapeutic potential of these cell death forms has also been recognized because they represent a promising alternative option to eliminate apoptosis-resistant cancer cells.

Interestingly, ferroptosis can be modulated by specific signalling pathways, and increasing evidence points to an important role of cell–cell contacts in regulating ferroptosis. Before we summarize and discuss the emerging concept on the role of cell–cell contacts in ferroptosis regulation, we first need to provide an overview on what is the current knowledge on the molecular mechanisms and characteristics underlying ferroptosis. 

## 2. Ferroptosis: Characterization and the Core Machinery

Ferroptosis is defined by two characteristic hallmarks, which are excessive lipid peroxidation and iron dependency. Ferroptosis lacks typical features of previously recognized cell death forms, such as apoptosis, autophagy, necroptosis, and oxidative stress-induced necrosis and was shown to operate in the absence of caspase activation, autophagic vesicle formation, RIPK3-dependency, or cytoplasmic swelling, respectively [4]. Morphologically, ferroptotic cells display condensed mitochondria and disruption of the outer mitochondrial membrane and finally undergo cell shrinkage. 

### 2.1. Lipid Peroxidation

Healthy cells maintain an equilibrium between permanently occurring lipid peroxidation and detoxification processes, thereby keeping the overall level of lipid hydroperoxides low. Ferroptosis is triggered by an imbalance between production and removal of lipid hydroperoxides in favour of their production. In most cases described, ferroptosis is induced by inhibition of detoxification processes. However, it can also be triggered by an increase in lipid peroxidation. Typically, lipid peroxidation of esterified fatty acids—i.e., phospholipids of cellular membranes, but not of free fatty acids—is required for ferroptosis [5]. Two enzymes are of main importance for ferroptosis: the acyl CoA-synthetase long chain family member 4 (ACSL4), which preferentially transfers arachidonic acid to acetyl-CoA and which is required for subsequent incorporation of the fatty acid into phospholipids by lysophosphatidylcholine acyltransferase 3 (LPCAT3) [5,6]. At least in some cell types, such as ovarian cancer cells, neurons, and cardiomyocytes, also formation of ether phospholipids appears to regulate ferroptosis [7]. 

Polyunsaturated fatty acids (PUFAs, >1 double bond), for instance alpha-linolenic and arachidonic acid, are especially prone to peroxidation. Monounsaturated fatty acids (MUFAs, one double bond) are much less susceptible to peroxidation, while saturated fatty acids withstand peroxidation. In accordance, ferroptosis can be attenuated by exo-genous addition of MUFAs which are incorporated instead of PUFAs by ACSL3 into the phospholipids of the plasma membrane [8]. 

**Figure 1:**
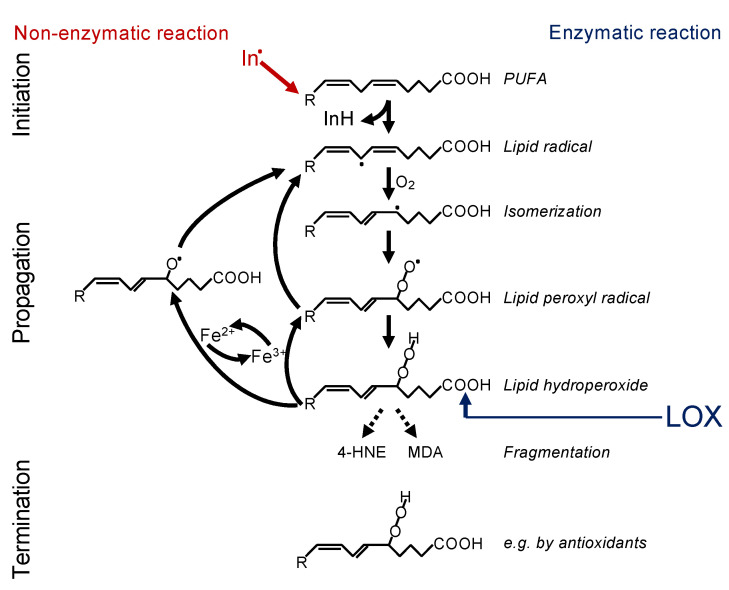
Mechanism of lipid peroxidation. Left: Radical initiation is carried out by a radical (initiator = In^.^) abstracting a hydrogen from a bisallylic site, i.e., the carbon between two double bonds of a PUFA, thereby creating a carbon-centred lipid radical (L*). Due to the adjacent double bonds, the radical can delocalize its electron system, resulting in a resonance-stabilized conformation. In a fast reaction, oxygen is then added, and a lipid peroxyl radical (LOO*) is formed. By abstracting a hydrogen from an adjacent PUFA, the lipid peroxyl radical itself reacts to a lipid hydroperoxide (LOOH) and generates the next carbon-centred radical (L*), thereby propagating the chain reaction. The lipid hydroperoxide (LOOH) is readily decomposed, either by ferrous iron (Fe^2+^) to the lipid alkoxyl radical (LO*) or by ferric iron (Fe^3+^) back to the lipid peroxyl radical (LOO*). Both radicals can fuel the chain reaction. The reaction is terminated when two radicals meet each other, e.g., forming a lipid dimer (L-L) or a peroxide-bridged lipid dimer (LOOL) (not shown), or when a radical meets a radical trapping agent, for instance, coenzyme Q10 or alpha-tocopherol (vitamin E), creating a lipid hydroperoxide (LOOH). Right: Alternatively, lipid peroxidation is carried out in a controlled manner by lipoxygenases (LOX). Although initiating the process at the level of lipid hydroperoxide formation, the lipid hydroperoxide will be decomposed by ferrous or ferric iron to lipid alkoxyl and peroxyl radicals, respectively, and the vicious circle of radical formation will be fuelled as described above. Hence, the initial enzymatic reaction is switched in a second step to a radical process [29].

In principle, there are two ways in which lipid peroxidation is triggered—i.e., non-enzymatically or enzymatically (Figure 1). Non-enzymatic lipid peroxidation is driven by radicals. Free electrons might emerge from leakage of electrons from the electron transport chain (ETC) in the inner mitochondrial membrane or might be released by enzymatic systems, such as NADPH oxidases (NOX)—e.g., NOX4 or NOX2 [4,9,10]. Such electrons can react with oxygen to form superoxide, which is converted to hydrogen peroxide by the superoxide dismutase. Under catalysis of ferrous iron (Fe^2+^), hydrogen peroxide reacts to the hydroxyl radical, a process known as the Fenton reaction. The hydroxyl radical itself, or rather an iron(IV)-oxo (ferryl) intermediate formed within the reaction, are highly reactive and may abstract a hydrogen from the bisallylic site of a PUFA [11,12]. This creates a lipid radical, known as initiation, and this lipid radical will lead to a chain reaction, referred to as propagation. The reaction will be terminated by an antioxidant or when two radicals meet [13]. In addition, fragmentation of the lipid hydroperoxides leads to the formation of secondary products, the most important of which are 4-hydroxynonenal (4-HNE) and malondialdehyde (MDA). 4-HNE is supposed to be the most toxic, while MDA appears to be the most mutagenic product of lipid peroxidation because it can efficiently attack DNA [14].

Recently, two groups independently discovered an important role of cytochrome P450 oxidoreductase (POR) in lipid peroxidation although the two molecular mechanisms proposed are rather contradictory [15,16]. While Yan and coworkers demonstrate an elevation of hydrogen peroxide and, in line, inhibition of lipid peroxidation by catalase, Zou and coworkers could not modulate sensitivity towards ferroptosis by expression of catalase, and the authors postulate POR-mediated formation of an iron-oxo species [16]. Thus, additional research efforts are required to solve these open questions.

Oxidation of lipids can also be started in a controlled manner by enzymatic processes. The most important enzymes that are capable of initiating lipid oxidation belong to the family of lipoxygenases (LOXs), which dioxygenate PUFAs to the lipid hydroperoxides (Figure 1). Cyclooxygenases are generally not involved in ferroptosis [17]. However, upregulation of COX2 (also known as PTGS2) is frequently observed and can be used as a marker for ferroptosis [18]. A potential role of cytochrome P450 in enzymatic lipid peroxidation during ferroptosis remains to be explored. 

**Figure 2:**
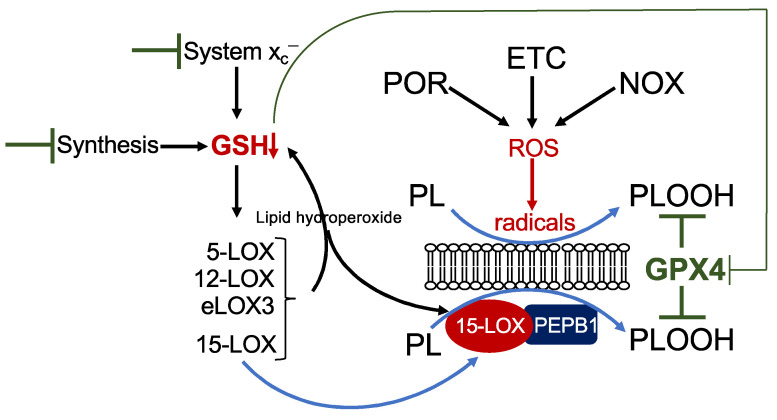
Mechanism of lipid peroxidation. Hypothetical model of phospholipid peroxidation leading to ferroptosis. Phospholipid peroxidation may be triggered either by radical formation due to reactive oxygen species (ROS) derived from cytochrome P450 oxidoreductase (POR), the electron transport chain (ETC), or NADPH oxidase (NOX), or enzymatically by 15-lipoxygenase (15-LOX). Association of 15-LOX with PEPB1 alters its substrate specificity towards membrane-bound PUFAs. Reduction in GSH by any means will increase the redox tone of the cell and thereby activate any LOX isoform. While 15-LOX can directly dioxygenate membrane-bound PUFAs, other isoforms indirectly cause accumulation of phospholipid hydroperoxides (i) by their primary reaction products, which activate 15-LOX, and (ii) by decreasing GSH levels, thereby attenuating GPX4 activity.

There are six isoforms of LOXs in humans: 5-LOX (gene *ALOX5*), 15-LOX1 (also 12/15-LOX, gene *ALOX15*), 15-LOX2 (gene *ALOX15b*), pl12-LOX (gene *ALOX12*), 12R-LOX (gene *ALOX12B*), and eLOX3 (gene *ALOXE3*) [19]. LOX enzymes are non-heme iron-containing enzymes, and the Fe^2+^ in the catalytic centre requires oxidation to Fe^3+^ for LOX activation. This may be facilitated by their own primary reaction products as well as a decrease in intracellular GSH [20]. Among all isoforms, 15-LOX has the highest activity towards esterified PUFAs in phospholipids [19,21]. Moreover, a small scaffolding protein, phosphatidylethanolamine-binding protein 1 (PEPB1), forms a complex with 15-LOX and enables 15-LOX activity on phospholipid-esterified PUFAs [22,23]. However, the specific role of LOX enzymes in ferroptosis is still a mystery and is highly context specific. Analysis is hampered by the fact that most of the LOX-specific inhibitors are also radical trapping agents and hence unsuitable for discriminating between a LOX-dependent or LOX-independent mechanism [24]. Focussing on those studies using loss-of-function analysis by genetic deletion or RNA interference, several aspects emerge. The contribution of LOX is cell-type specific. While in the fibrosarcoma cell line HT-1080, 15-LOX1 is required for erastin- and RSL3-triggered ferroptosis [25], other cell lines which do not express any LOX isoform are still fully sensitive to erastin (own unpublished observation). Hence, other factors, such as POR-, NOX-, and/or ETC-derived radicals very likely contribute to lipid peroxidation (Figure 2). Furthermore, the role of LOX depends on the ferroptotic stimulus. Although mechanistically not understood, 12-LOX is involved in p53/ROS-, but not erastin-induced ferroptosis in osteosarcoma cells [26]. Simultaneous downregulation of all six LOX isoforms in a human kidney rhabdoid tumour cell line results in inhibition of erastin-, but not of RSL3-triggered ferroptosis [27]. In line with this observation, mouse embryonic fibroblasts derived from *Alox15* knock-out mice are highly resistant to buthionine-sulfoximine (BSO)-mediated GSH depletion [17], but knock-out of *Alox15* in fibroblasts derived from *Gpx4* knock-out mice does not protect against ferroptosis [28]. The fact that LOX activation is coupled to the cellular redox state including to GSH levels provides a reasonable explanation to reconcile the above-described discrepancies. Mechanisms leading to a decrease in GSH, such as inhibition of GSH synthesis by BSO or inhibition of cystine uptake by erastin, will lead to an inevitable activation of LOX, whereas pharmacological inhibition by RSL3 or genetic depletion of *GPX4* is not sufficient to result in LOX activation (Figure 2). Finally, the contribution of LOX may even vary between sub-cell lines. As outlined above, Shintoku and coworkers identified 15-LOX1 as the pivotal LOX in ferroptosis in HT-1080 cells. In contrast, Yang and coworkers solely detected 15-LOX2 and eLOX3 in HT-1080 cells. However, downregulation of either isoform provided resistance to erastin-mediated ferroptosis, although only free, but not esterified PUFAs, are eLOX3 substrates [27]. In principle, it may be assumed that free lipid peroxides derived from any LOX isoform may finally activate 15-LOX in the direct vicinity, which then in turn dioxygenates phospholipid-esterified PUFAs [20]. In addition, activation of any LOX isoform will cause a reduction in GSH due to GPX4-mediated detoxification of the lipid hydroperoxides (Figure 2). Hence, LOX enzymes appear to be required for ferroptosis in some but not all circumstances and might be dependent on the cellular context and the expression of specific LOX isoforms and/or the ferroptotic stimulus, which can trigger different routes to ferroptosis. 

### 2.2. Iron Dependency

Iron homeostasis in a cell is tightly regulated by import, storage, and export (for detailed overview see [30,31,32]). The most important transporter for iron intake is the transferrin receptor 1 (TfR1). Extracellular transferrin, which carries two ferric ions, binds to the TfR1 in the plasma membrane, and the complex is then internalized by endocytosis. Endosomes are acidified forming lysosomes, and ferric iron is reduced to ferrous iron by proteins of the STEAP family, which causes a release of iron from transferrin. Hence, lysosomes contain an important pool of intracellular iron. Ferrous iron can cross the endosomal membrane mediated by the divalent metal-ion transporter 1 (DMT1). Cytoplasmic iron is transferred to the storage protein ferritin, or, e.g., used for the synthesis of iron-containing enzymes. Ferritin is composed of a light (FtL) and a heavy chain (FtH), the latter being endowed with ferroxidase activity, thereby oxidizing ferrous to ferric iron, hence preventing Fenton reaction and intracellular ROS formation. Interestingly, a second protective function of FtH might be inhibition of epithelial–mesenchymal transition [33], a process affecting ferroptosis as described below. A small pool of iron resides in the cytoplasm, either as free iron or bound to chaperones, such as poly(rC)-binding proteins, thereby forming the so-called labile iron pool (LIP) [34]. Excess of intracellular iron leads to its export by the transporter ferroportin 1. 

The precise role of iron in ferroptosis has not been elucidated so far [35]. The conclusion that iron is absolutely required for ferroptosis comes from the observation that iron chelators inhibit ferroptosis [4,36,37]. In accordance, decreasing the LIP, e.g., by increased expression of ferritin, suppresses ferroptosis [38]. Vice versa, intracellular increase in LIP, e.g., by lysosomal destabilization or ferritinophagy, supports ferroptosis [39]. In principle, three possibilities can be considered to explain this finding: (i) iron is present as a cofactor in the active centres of enzymes, (ii) iron acts as an inducer of lipid peroxidation, and (iii) iron functions as a mediator of propagation. 

### 2.3. Detoxification Processes

Three main antioxidant mechanisms regulating ferroptosis have been revealed: (i) the GPX4/GSH, (ii) the FSP1/CoQ10, and (iii) the GCH1/BH_4_ system. Among the various isoforms, the selenoprotein GPX4 is the only one which—with its cofactor GSH—directly detoxifies lipid hydroperoxides by promoting the reaction to lipid alcohols [40]. The synthesis of GSH is dependent on the availability of cysteine, which is derived from its precursor molecule cystine. Cystine is taken up by specific amino acid transporters; an important one is system x_c_^−^. System x_c_^−^ is composed of two subunits, the light chain SLC7A11 and the heavy chain SLC3A2, and imports extracellular cystine in exchange of intracellular glutamate [41]. The importance of the GPX4/GSH detoxification mechanism in preventing ferroptosis is demonstrated by the fact that genetic deletion or pharmacological inhibition of GPX4 as well as downregulation of its cofactor GSH by any means will cause an accumulation of lipid hydroperoxides and frequently results in ferroptosis [4,18,28].

A second important detoxification system, which acts in parallel to GPX4/GSH, is the FSP1/CoQ10 system. CoQ10 is well-known for its electron shuttling role in the ETC in the mitochondria, but it also plays an essential role as a lipid radical trapping agent. It is composed of a polar head containing a redox reactive benzoquinone ring and a tail composed of ten isoprenoid units, which anchors the enzyme in the lipid bilayer of biological membranes [42]. The isoprenoid moieties used for CoQ10 synthesis are provided by the mevalonate pathway. Mechanistically, the reduced form of CoQ10 (CoQ10H_2_) can successively trap two electrons, thereby preventing radical initiation as well as propagation of lipid peroxidation by scavenging lipid peroxyl radicals. A second function is to regenerate alpha-tocopherol from the tocopheroxyl radical, another important physiological lipid antioxidant [43]. Two independent studies found that the FSP1/CoQ10 system provides resistance to ferroptosis [44,45]. FSP1, ferroptosis suppressor protein 1 (also known as apoptosis-inducing factor mitochondria-associated 2, AIFM2), is recruited to the plasma membrane by myristoylation. Here, it functions as an oxidoreductase which—together with its cofactor NAD(P)H—reduces CoQ10, thereby providing new functional CoQ10H_2_. However, genetic deletion or pharmacological inhibition of FSP1 is not sufficient for inducing ferroptosis, but it sensitizes to ferroptosis triggered by inhibition of GPX4 or GSH synthesis.

In a CRISPR activation screen, a third independent protective pathway has been identified involving the GTP cyclohydrolase 1 (GCH 1), which is responsible for the synthesis of tetrahydrobiopterin (BH_4_) [46]. In addition to other functions, BH_4_ acts as a direct radical scavenger [12]. It can interfere with lipid peroxidation at the level of initiation and propagation [47]. Thereby, BH_4_ provides a powerful protection against ferroptosis induced by GPX4 depletion, GPX4 inhibition, or GSH downregulation [46]. Because BH_4_ is also required for CoQ10 synthesis, it additionally acts by increasing CoQ10 levels. For unknown reasons, BH_4_ specifically prevents degradation of phospholipids containing two PUFAs. To note, BH_4_ is a diffusible molecule and therefore able to protect surrounding cells [46]. Although overexpression of GCH1 clearly provides resistance to ferroptosis, even in GPX4-depleted cells, deletion of GCH1 is not sufficient for spontaneously triggering induction of ferroptosis [46].

### 2.4. Inducers of Ferroptosis

In principle, ferroptosis can be induced by the canonical pathway, which is inhibition of the GPX4/GSH defence pathway, or, non-canonically, by direct stimulation of lipid per-oxidation (for detailed overview see [48]). Canonical inducers comprise class I to class III ferroptosis inducers (FIN). 

**Figure 3:**
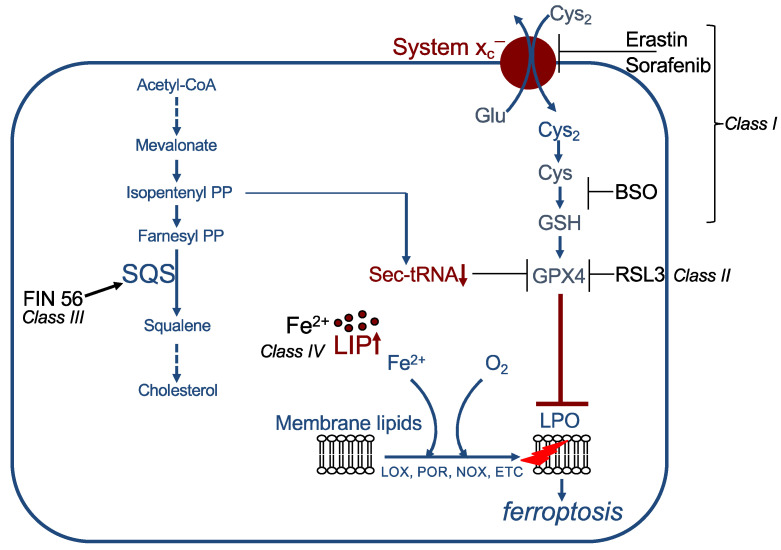
Examples of ferroptosis inducers (FINs). Class I FINs act by decreasing GSH levels. Class II FINs directly inhibit GPX4. Class III FINs indirectly block GPX4 activity. Class IV FINs increase the labile iron pool (LIP). See text for details. SQS: squalene synthase, LIP: labile iron pool, LPO: lipid peroxidation, PP: pyrophosphate.

The so-called class I inducers indirectly inhibit GPX4 activity by depletion of its cofactor GSH via decrease in its synthesis (Figure 3). This can be achieved, for instance, (i) by blocking system x_c_^−^, thereby reducing cystine uptake (important examples are erastin, sorafenib, sulfasalazine, or excess of glutamate); (ii) by inhibiting glutamate cysteine ligase, e.g., with BSO; or (iii) by enzymatically degrading cyst(e)ine, carried out by cyst(e)inase [4,18,49,50,51,52]. Class II inducers directly block GPX4 activity. The most studied compound is RSL3, which covalently binds to the selenocysteine in the active site of GPX4 [18,37]. Additional examples are ML210 and altretamine [53,54,55]. Class III inducers, e.g., FIN56, indirectly block GPX4 activity. FIN56 binds to and activates the enzyme squalene synthase. Squalene synthase is required for cholesterol synthesis and catalyses the reaction from farnesyl pyrophosphate to squalene. This reaction leads to consumption of farnesyl pyrophosphate and its precursor isopentenyl pyrophosphate. Because farnesyl pyrophosphate is also a precursor of CoQ10 (see above), this might lead to a reduction in CoQ10 levels. More important, isopentenyl pyrophosphate is required for selenocysteine-tRNA functionality. Downregulation of isopentenyl pyrophosphate thus results in loss of GPX4 activity [56]. The so far identified non-canonical inducers, also referred to as class IV inducers, directly stimulate lipid peroxidation by an increase in the LIP. This can be caused by different mechanisms, (i) by addition of exogenous FeCl_2_, (ii) by changing iron uptake and export, e.g., by combination of lapatinib and siramesine, (iii) by lysosomal destabilization, or (iv) by degradation of heme oxygenase 1 (HO1) [55,57,58,59]. 

Some ferroptosis inducers have multiple targets: withaferin A, a steroidal lactone derived from some solanaceae, belongs to class II inducers and—as a class IV inducer—leads to degradation of HO1 [55]. The endoperoxide FINO2 indirectly blocks GPX4 function and can be regarded at the same time as class IV inducer due to its iron oxidating function [60]. Furthermore, the recently characterized ferroptosis activator talaroconvolutin A, a natural compound isolated from the endophytic fungus *Talaromyces purpureogenus*, leads to deregulation of various ferroptosis-related genes, such as downregulation of the cystine transporter component SLC7A11, upregulation of LOXE3 and LOX12, as well as an increase in several iron-metabolism-related genes in colorectal cancer cells [61]. 

It is still a matter of debate whether the addition of exogenous ROS might trigger ferroptosis. Our findings demonstrate that the organic *tertiary*-butyl hydroperoxide (*t*-BuOOH) is a potent ferroptosis inducer in murine and human cell lines [62]. Similar observations were made by Jiang and coworkers, although upregulation of p53 was required for *t*-BuOOH-triggered ferroptosis in their cellular model [63]. Taken together, there are a multitude of different ferroptosis activators which initiate ferroptotic cell death through canonical and non-canonical mechanisms.

### 2.5. Inhibitors of Ferroptosis

Ferroptosis is driven by iron-dependent lipid peroxidation, which takes place in phospholipids, and—independent from the initial trigger—is carried out in a second step by a radical chain reaction. In consequence, ferroptosis induction can be counterbalanced by antioxidants, and, in addition, can be pharmacologically attacked at any of these levels. Among the various compounds known to inhibit ferroptosis, the most typical ones need to be mentioned in the following. As already outlined above, iron-chelators, such as deferoxamine and ciclopirox, are powerful inhibitors of ferroptosis [4]. Lipid peroxidation can efficiently be blocked by the radical trapping agents ferrostatin 1 and liproxstatin 1 as well as the endogenous antioxidant alpha-tocopherol (vitamin E) [4,28,64,65]. Increasing the cellular antioxidative activity by idebenone, a synthetic and soluble analogue of CoQ10, also attenuates ferroptosis [44]. It has further been demonstrated that thiazolidinediones decrease ferroptosis by blocking ACSL4. In general, ferroptosis inhibition could be of relevance in specific pathophysiological settings including neurodegenerative diseases, which may also include ferroptotic cell death. For a more comprehensive description of inhibitors, the reader is referred to some recent overview articles [66,67,68,69].

### 2.6. Execution of Ferroptosis

Ferroptosis is executed by nanopore formation; influx of extracellular ions, including calcium; influx of water, cell swelling, and membrane rupture; and finally, cell shrinkage [70]. Cells try to counterbalance the lytic damage by activating the calcium-dependent endosomal sorting complex required for transport (ESCRT-III) machinery [71]. This hetero-multimeric protein complex is able to repair damaged membranes by scission and sealing [72]. Interestingly, the cellular compartment or organelle in which lipid peroxidation takes place is still an open question. In addition, the mechanism by which the signal is transduced to the plasma membrane and what causes pore formation still remain elusive. The simplest explanation would be direct toxicity of phospholipid hydroperoxides and/or lysophospholipids derived thereof—for instance, by disorientation of peroxidized phospholipids and conformational changes in the plasma membrane [73]. However, several observations argue for a specific role of a subset of phospholipids in ferroptosis. Per-oxidation of fatty acids has been detected in a broad range of phospholipids, and a broad spectrum of oxidized PUFAs is observed [55,74,75]. Peroxidized PUFAs are then enzymatically cleaved resulting in lysophospholipids, i.e., lysophosphatidylcholines and lysophosphatidylethanolamines. However, not all of them are required for ferroptosis [60]. Oxygenation of ω-6 fatty acids, such as arachidonic acid and its elongation product adrenic acid, in phosphatidylethanolamines especially seems to be of functional importance for driving ferroptosis [5,74]. Agmon and coworkers suggest that—due to their conic structure—oxidation of phosphatidylethanolamines would especially lead to severe membrane destabilization. They propose a model in which peroxidation of PUFA-containing phosphatidylethanolamines causes thinning of the plasma membrane, leading to increased access of oxidants, hence starting a vicious circle, finally ending with increased plasma curvature, miscellation, and pore formation [76]. Of note, lipid peroxidation in the plasma membrane can be seen in some [8,44,45,71] but not all cases. Lipid peroxidation was also observed in the mitochondria and at later time points in the plasma membrane [77], the endoplasmatic reticulum, the lysosomes, or Golgi complex [25,39,78,79]. Hence, some currently undefined downstream signalling events also have to be postulated. Reasonable candidates are lipid hydroperoxide-derived aldehydes, such as 4-HNE and MDA, which readily form adducts with proteins and DNA [80]. Indeed, protein-carbonylation could be detected during ferroptosis [81]. It is noteworthy, that membrane permeabilization due to photosensitizer-mediated lipid peroxidation is connected with the presence of aldehydes [82]. In line with this observation, high expression of aldo-keto reductases, which detoxify aldehydes including 4-HNE, provide resistance to ferroptosis [78,83]. Finally, the involvement of lysosomal enzymes cannot be ruled out as playing a role in ferroptosis [84]. 

Having provided a broad overview on the cellular and biochemical core machinery initiating, modulating, and executing ferroptotic cell death in the previous chapter, we now summarize and discuss how ferroptosis commitment is regulated by the cellular context and in particular by cell–cell contacts, which frequently differs upon physiological and pathophysiological conditions. For a detailed description of other regulatory mechanisms, such as degradation systems, transcription factors, or epigenetic regulators, the reader is referred to excellent reviews [48,85,86,87].

## 3. Regulation of Ferroptosis by Cell–Cell Contacts

### 3.1. Cell–Cell Contacts, Adherens Junctions, and Cadherins

Cell–cell contacts regulate embryonic development and are crucial for the maintenance of tissue homeostasis in the adult organism. Beyond their role in mechanical intercellular adhesion and tissue barrier function, they contribute to signalling events and control cellular proliferation, survival, differentiation, and migration. 

There are different types of cell–cell contacts, especially in epithelial cells, which connect a cell to neighbouring cells. These cell–cell contacts include adherens junctions, tight junctions and desmosomes [88]. However, also non-epithelial cells, such as fibroblasts, express similar adherens and tight junction structures [89,90]. In epithelial cells, adherens junctions are built up by E-cadherin dimers, which bind to another E-cadherin dimer on the neighbour cell in a homophilic, calcium-dependent manner. The cytoplasmic domain of E-cadherin is linked to the actin cytoskeleton by a complex consisting of p120, α-, β-, and/or γ-catenin. One important function of E-cadherin is to inhibit proliferation, also referred to as “contact-dependent inhibition of proliferation” or “contact inhibition” [91]. Contact inhibition is active in the adult tissues of an organism, and it is a well-known phenomenon seen in 2D cell culture, in that non-transformed cells are arrested in G0/G1-phase when they have reached a critical cell density and form a confluent monolayer [92,93,94,95,96,97,98].

In contrast to E-cadherin, N-cadherin is a mesenchymal marker and can exert a pro-invasive role when aberrantly expressed during epithelial–mesenchymal transition [99]. However, N-cadherin may also promote adhesion, induce contact inhibition, and regulate differentiation [100,101,102]. Similar to E-cadherin, N-cadherin forms calcium-dependent homophilic interactions, is linked via α-catenin to the cytoskeleton, and recruits β-catenin to the plasma membrane. It is expressed in a cell-type specific way in the adult organism—for instance, in neurons, fibroblasts, endothelial cells, and myocytes [102]. N-cadherin represents a major cell adhesion molecule also in vitro—for example, in fibroblasts [103,104] (own unpublished observations).

### 3.2. A Role of Adherens Junctions and E-Cadherin in Growth Control and Differentiation

E-cadherin is important for establishing epithelial cell differentiation and cell polarity, which are critical for the function of epithelial cell layers. Furthermore, E-cadherin prevents epithelial–mesenchymal transition (EMT), a biological process essentially contributing to the dedifferentiation and carcinogenesis of epithelial cells. During EMT, epithelial cells lose their cell contacts, their characteristic cobblestone morphology, and their polarity to acquire a mesenchymal phenotype supporting migration and metastasis (for detailed description see [105,106,107]). Three core transcription factors are involved in EMT—i.e., SNAI1/SNAI2 (formerly known as Snail/Slug), TWIST, and ZEB1/2, which repress epithelial markers including E-cadherin transcription. As a consequence, mesenchymal markers, such as fibronectin, vimentin and N-cadherin, are upregulated [108]. 

**Figure 4:**
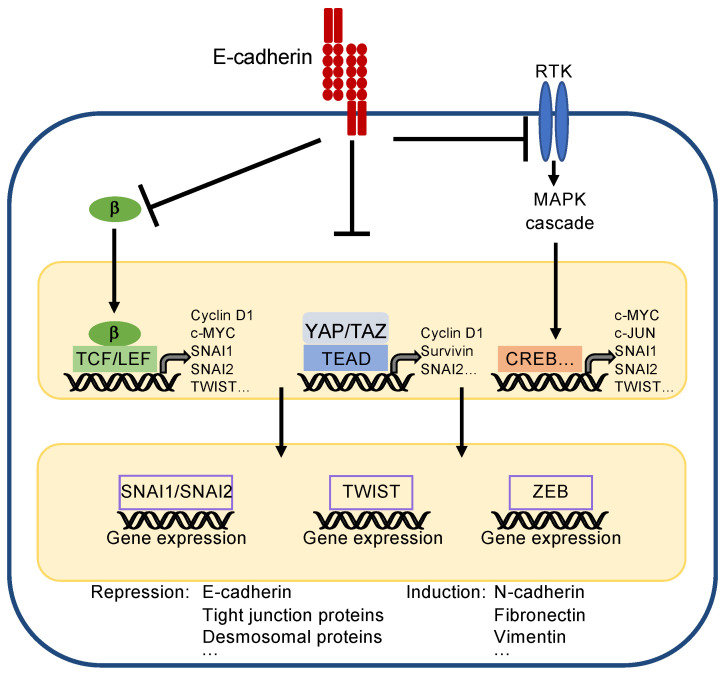
Simplified scheme to present principles of EMT. The core transcription factors inducing EMT are SNAI1/SNAI2, TWIST, and ZEB. They reduce expression of epithelial markers, such as E-cadherin, and induce expression of mesenchymal markers, such as N-cadherin. They are regulated by upstream acting transcription factors, which also support proliferation, survival, and EMT by their own gene expression. (Note: CREB is representative for all transcription factors activated by the MAPK cascade). E-cadherin inhibits EMT by recruiting β-catenin (β), sequestering YAP/TAZ, and by inhibiting activity of receptor tyrosine kinases (RTKs), which, e.g., activate the mitogen-activated protein kinase (MAPK) cascade.

The inhibition of proliferation and maintenance of epithelial differentiation is achieved by suppression of several master pathways and regulators, such as the Wnt pathway, receptor tyrosine kinases (RTK) activating the mitogen-activated protein kinase (MAPK) and PI3K/AKT pathway, and the transcriptional coactivators YAP/TAZ (yes-associated protein/transcriptional co-activator with PDZ-binding motif) [106,109]. One important function of E-cadherin is to sequester β-catenin, thereby keeping the level of cytoplasmic β-catenin low (Figure 4). β-catenin is a core component of the Wnt pathway [105,110]. Accumulation of β-catenin in the cytoplasm, which occurs physiologically during embryonic development and pathologically during tumourigenesis, leads to its nuclear translocation, binding to transcription factors of the TCF/LEF (T-cell factor/lymphoid enhancer factor) family and transcriptional activation of genes controlling proliferation, such as *CCND1* (encoding for cyclin D1), *c-MYC*, and *c-JUN*. Moreover, TCF/LEF factors can directly repress E-cadherin (gene *CDH1*) transcription and activate EMT-inducing transcription factors, such as *SNAI* and *TWIST* [111]. 

Inhibition of RTK signalling can, for instance, be achieved by direct binding of E-cadherin to RTKs, such as the epidermal growth factor receptor (EGFR), and can attenuate their kinase activity, or indirectly via the tumour suppressor neurofibromatosis type 2 (NF2 or merlin) [112] (Figure 4). Attenuation of RTK signalling leads to decreased transcription of mitogenic genes, such as *c-MYC* and *c-JUN*, but also of the classical EMT transcription factors *SNAI, TWIST*, and *ZEB1*/*2* [113].

Finally, the inhibition of the transcriptional coactivators YAP/TAZ is crucial for contact inhibition (Figure 4), which is mediated by stimulation of the Hippo pathway [114,115] (for review see [112,116]). Although regulation of the Hippo pathway is complex, the Hippo core pathway basically consists of merlin and two Ser/Thr protein kinases, MST1/2 (mammalian STE20-like kinases) and LATS1/2 (large tumour suppressor), together with their regulatory subunits SAV1 (salvador 1) and 2-MOB1 (2-monopolar spindle-one-binder protein1), respectively, finally leading to phosphorylation and inhibition of YAP/TAZ by nuclear exclusion and protein degradation [116]. In their active state, YAP/TAZ bind to transcription factors of the TEAD (transcriptional enhancer factor domain) family resulting in transcriptional activation of genes involved in epithelial–mesenchymal transition, cell proliferation, migration, antiapoptotic mechanisms, stem cell properties, and metabolism. Critical TEAD target genes are *CCND1* (encoding for cyclin D1), which facilitates cell proliferation, the EMT regulator *SNAI2* [117], or the anti-apoptotic gene *BIRC5* (encoding for survivin) (for a detailed description of YAP/TAZ function see [118,119,120]). 

### 3.3. How Do Cadherins Crosstalk to the Hippo Pathway?

**Figure 5:**
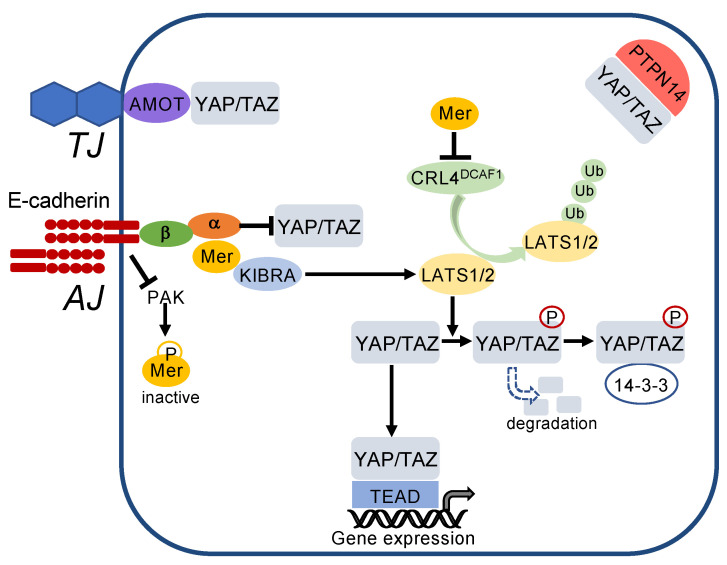
Cell–cell contacts turn on the Hippo pathway. E-cadherin inactivates p21-activated kinase (PAK), thereby blocking phosphorylation and inactivation of merlin (Mer). Active merlin associates with KIBRA and activates LATS1/2, which phosphorylates YAP/TAZ. This leads to cytosolic sequestration and degradation of YAP/TAZ. In addition, merlin inhibits activity of the E3 ubiquitin ligase CRL4^DCAF1^ thereby blocking degradation of LATS1/2. YAP/TAZ are also directly sequestered to adherens junctions (AJ) by α-catenin (α), which is associated via β-catenin (β) with E-cadherin. Moreover, YAP/TAZ are bound to tight junctions (TJ) via angiomotin (AMOT) and sequestered in the cytoplasm by the protein tyrosine phosphatase nonreceptor (PTPN)14.

E-cadherin regulates the Hippo pathway at multiple levels (Figure 5). E-cadherin mediates suppression of PAK (p21-activated kinase) activity and thereby favours the dephosphorylated, active state of merlin [121]. Merlin associates with KIBRA and in turn phosphorylates LATS1/2, resulting in phosphorylation of YAP/TAZ. Merlin also inactivates the E3 ubiquitin ligase CRL4^DCAF1^, thereby preventing LATS1/2 degradation. As a result, phosphorylated YAP/TAZ are excluded from the nucleus and bind to the cytosolic scaffold protein 14-3-3. In addition to retention in the cytosol, phosphorylation of YAP/TAZ leads to their proteasomal degradation [122]. YAP/TAZ are also directly sequestered by binding to α-catenin at the adherens junctions (Figure 5). Independent from E-cadherin, YAP/TAZ bind to AMOT (angiomotin) at tight junctions and are sequestered in the cytosol by the protein tyrosine phosphatase nonreceptor (PTPN)14 [121,123,124,125]. 

Having provided the essential background information on cell–cell contacts and their role in growth control, we now summarize and discuss their function in ferroptosis regulation.

### 3.4. Cell–Cell Contacts as Regulators of Ferroptosis

Interestingly, recent observations have demonstrated a significant role of cell–cell contacts, in particular adherens junctions, in controlling ferroptosis. This fascinating link was not immediately expected because it is widely accepted that E-cadherin signalling especially supports apoptosis and, vice versa, EMT protects cancer cells against apoptosis, correlating with a poor clinical prognosis [126,127,128]. 

Observations from the early 1960s indicated that cells lose sensitivity to cysteine depletion with increased cell-density [129]. Later, it was shown that mouse embryonic fibroblasts with inducible *GPX4* depletion are protected against cell death at high cell densities [17], suggesting a role of cell–cell contacts on ferroptosis sensitivity. However, a specific role of cell–cell contacts in regulating ferroptosis has been shown only recently. Using several murine and human cell lines, we demonstrated that cell–cell contacts protect against ferroptosis induced by erastin or the organic *tertiary*-butyl hydroperoxide (*t*-BuOOH) [130]. Basal as well as induced lipid peroxidation was strongly reduced in confluent cells. Notably, this protective mechanism occurs independently from contact inhibition because the cells were seeded below their saturation density, thus ensuring cell–cell contact formation but excluding growth inhibition and quiescence at the time of treatment. Interestingly, cell–cell contacts also provide resistance to *t*-BuOOH-mediated loss of mitochondrial membrane potential and DNA double-strand break formation, but they failed to prevent *t*-BuOOH-triggered replication blockade or the formation of the oxidative base lesion 8-oxo-dG. These findings indicate that cell–cell contacts confer a broader, selective protection against cellular oxidative stress. Moreover, resistance was also observed in response to treatment with hydrogen peroxide, methyl methanesulfonate, or UV-C [130]. Hence, it is reasonable to conclude that protection by cell–cell contacts is more widespread than hitherto expected. 

**Figure 6:**
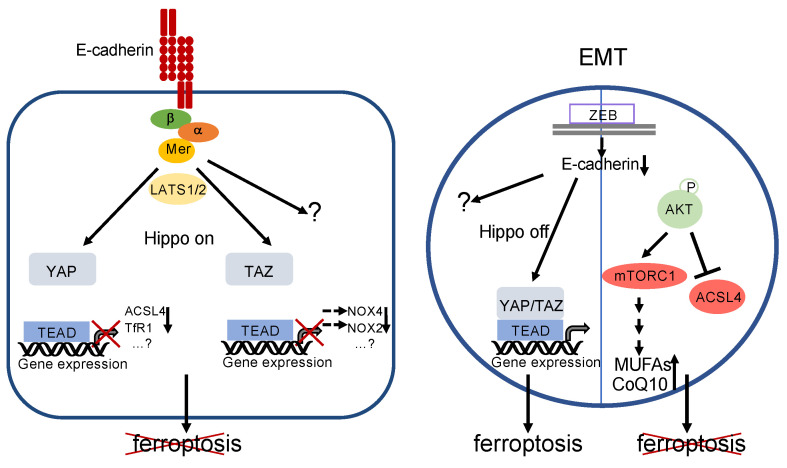
Regulation of ferroptosis by E-cadherin and EMT. Left: E-cadherin activates the Hippo pathway, thereby inactivating YAP- and TAZ-activity. This leads directly or indirectly to decreased expression of proteins that are relevant for ferroptosis (note that decreased levels of NOX4 and NOX2 are due to reduced expression of *EMP1* and *ANGPTL4*, respectively, see text for details). However, involvement of additional genes and/or signalling pathways is likely. Ferroptosis is blocked. Right: EMT—for instance, triggered by activation of ZEB—leads to a loss of E-cadherin expression and inhibition of the Hippo pathway. Although the downstream targets have not been elucidated so far, YAP/TAZ activation supports ferroptosis. Additional pathways remain to be elucidated. However, this can be counterbalanced by phosphorylation of AKT (or, possibly, other mechanisms). Activated AKT may lead to a decreased level of ACSL4 as well as increased formation of MUFAs and CoQ10 via mTORC1 stimulation. Ferroptosis is prevented.

Elegant work by Wu and coworkers identified the Hippo pathway as a crucial mediator of resistance to ferroptosis [131] (Figure 6). Using a colon carcinoma cell line and several genetic gain-of-function as well as loss-of-function analyses, the authors demonstrate that, in confluent cultures, E-cadherin leads to nuclear extrusion of YAP via PAK–merlin–LATS1/2 signalling (see above). Inactivation of YAP results in transcriptional downregulation not only of canonical YAP target genes but also of the iron-controlling TfR1 (encoded by the *TFRC* gene) and of ACSL4. A similar protective pathway seems to be induced by N-cadherin in fibroblasts and mesothelioma cells [131]. However, because co-overexpression of TfR1 and ACSL4 could not fully restore ferroptosis in these cells, additional ferroptosis-regulating target genes have to be postulated [131]. Moreover, very recently, it has been shown that YAP may regulate ferroptosis via induction of the E3 ubiquitin ligase SKP2, possibly by a positive feedback loop via nonproteolytic polyubiquitination, thereby maybe leading to enhanced YAP-TEAD interaction [132]. However, a proteolytic role of SKP2 on an unidentified substrate controlling ferroptosis cannot be formally excluded yet.

It has also been demonstrated that the YAP paralogue TAZ impacts on ferroptosis. In renal cell carcinoma, TAZ stimulates the expression of the epithelial membrane protein 1 (EMP1), which in turn activates p38 MAPK and finally induces the accumulation of NOX4 [9]. In ovarian carcinoma cell lines, TAZ upregulates NOX2 expression via increased induction of ANGPTL4 (angiopoietin-like protein 4) [10]. Although in both publications ferroptosis was inhibited at high cell density, and a regulatory function of TAZ/NOX4 and TAZ/NOX2, respectively, at high cell density is likely, the authors did not demonstrate downregulation of these target genes at cell confluency. Furthermore, the upstream signalling of this effect remains to be elucidated. 

These observations raise the interesting question of whether a mesenchymal phenotype of carcinoma cells does also increase ferroptosis sensitivity and, if so, what would be the underlying mechanism. Indeed, recurrent breast cancer cells having acquired a mesenchymal phenotype show upregulation of the receptor for collagen I discoidin domain receptor tyrosine kinase 2 (DDR2), which causes activation of YAP/TAZ and increases ferroptosis susceptibility [133]. The authors also provide evidence that induction of EMT in epithelial breast cancer cells by overexpressing TWIST or SNAI1 leads to similar results [133]. In accordance, a reciprocal association between differentiation and sensitivity to ferroptosis has been shown in a panel of head and neck cancer cells with either epithelial or mesenchymal phenotype [134]. Inducing EMT by different means, such as downregulation of E-cadherin, overexpression of ZEB1, or TGFβ1 treatment (which induces SNAI1, TWIST, and ZEB1/2), increases the cellular sensitivity to ferroptosis. Conversely, activation of mesenchymal–epithelial transition by restoring E-cadherin expression, inactivation of ZEB1, or epigenetic reprogramming leads to ferroptosis resistance. A similar correlation was observed in pancreatic cancer cells in response to TGFβ1 treatment [135]. In a variety of therapy-resistant cancer cells exhibiting an intrinsic, acquired, or induced mesenchymal state, expression of ZEB1 was strongly associated with their susceptibility to ferroptosis triggered by GPX4 inhibition [136]. In contrast, induction of EMT by overexpression of SNAI1 or TWIST did not consistently lead to sensitization [137]. A possible explanation might be provided by the fact that ZEB1 is a lipogenic factor and thereby alters cellular lipid metabolism. 

A different mechanism is provided by increased expression of metadherin. Metadherin (also known as astrocyte elevated gene 1 or LYRIC) is a tumour-associated antigen that promotes EMT, including downregulation of E-cadherin by activating multiple pathways, such as Wnt/β-catenin, MAPK, and PI3K/AKT signalling [137]. Interestingly, metadherin also functions as an RNA-binding protein, leading to downregulation of GPX4 and SLC3A2, the system x_c_− dimerization partner, and consequently to an elevated vulnerability to ferroptosis inducers [138].

These observations argue against the hypothesis that EMT is per se sufficient to increase ferroptosis susceptibility. Indeed, ferroptosis sensitivity might be counterbalanced by the AKT signalling pathway (Figure 6). In gallbladder cancer cells, downregulation of the histone deacetylase sirtuin 3 leads to typical features of EMT and increased phosphorylation of AKT (at ser473). However, they are protected against ferroptosis by AKT-mediated reduction in ACSL4 [139]. Ferroptosis resistance might also be a result of activating PI3K mutations or loss of PTEN function, leading to sustained mTORC1 activity via the PI3K–AKT–mTOR pathway [140]. Sustained mTORC1 activity leads to increased transcriptional activity of the sterol regulatory element-binding protein 1 (SREBP1), resulting in increased expression of the stearoyl CoA desaturase 1 (SCD1) [140]. SCD1 is an enzyme which converts unsaturated fatty acids to MUFAs. Hence, it is likely that MUFAs are incorporated instead of PUFAs into the plasma membrane, thereby decreasing the rate of lipid peroxidation in response to ferroptotic triggers [8]. Furthermore, SCD1 induces CoQ10 synthesis, which also contributes to ferroptosis resistance [141].

## 4. Conclusions and Outlook

There is increasing evidence that cell–cell contacts regulate ferroptosis sensitivity and mediate resistance to ferroptosis. The E-cadherin/N-cadherin–merlin–YAP–TfR1/ACSL4 pathway has been identified as one important mediator. However, it is currently not known whether other downstream targets, other pathways, or other cell adhesion molecules also contribute to or antagonize ferroptosis protection. Considering the multitude of YAP/TAZ target genes and especially their role in metabolism, it is tempting to speculate that additional mechanisms contribute to E-cadherin–YAP/TAZ-mediated protection. For example, transcription of enzymes involved in glutaminolysis requires YAP/TAZ, and it is known that glutaminolysis supports ferroptosis [142]. Our own unpublished data even suggest E-/N-cadherin-mediated protection independent of YAP/TAZ (own unpublished observations). One interesting transcription factor at the intersection of EMT and ferroptosis might be BACH1. BACH1 promotes EMT by multiple mechanisms and has recently been shown to facilitate ferroptosis by downregulation of several genes which protect against ferroptosis [143,144,145]. Furthermore, other cell adhesion molecules might influence ferroptosis. For instance, merlin is also known to be regulated by tight junctions. Cell clustering by nectins provide resistance in detached breast and lung carcinoma cells under specific circumstances [146]. Moreover, gap junctions might play a role in regulating ferroptosis, possibly by facilitating diffusion of NADPH [147]. However, these findings should lead to a change in our perception that solid, differentiated tumours expressing E- or N-cadherin are generally prone to cell death. Although they might be susceptible to apoptosis, this might not be the case for ferroptosis. These observations may open new avenues for the treatment of some tumour entities with poor prognosis, i.e., those having acquired a mesenchymal and invasive phenotype, which is typically associated with high therapy resistance. Such tumours include pancreatic and diffuse gastric tumours, triple negative breast cancer, melanoma, recurrent tumour cells, and cancer stem cells [136,148,149]. Although first in vivo data are encouraging [52], still numerous important questions remain to be answered in the future. As outlined above, EMT is not sufficient per se, and more work is necessary to define intracellular signatures rendering tumour cells susceptible to ferroptosis. Another important aspect is that EMT in cancer progression is not a binary programme, but transitional states are postulated. Although not proven, in vitro and in vivo data suggest that EMT is initiated by the activation of SNAI1/2, whereas ZEB and TWIST are required for further transitions [150]. At which step does ferroptosis sensitivity occur and is this sensitivity dependent on the activation of a specific transcription factor? While Lin and coworkers could induce erastin sensitivity by overexpression of TWIST or SNAI1 in primary mouse breast tumour cells, overexpression of SNAI1 in MCF7 breast or of TWIST in a lung cancer cell line did not change susceptibility to GPX4 inhibition [133,136]. These discrepancies might be due to the different ferroptotic stimuli the authors used, or they might be due to gene mutations changing intracellular signalling and additional factors regulating EMT, such as miRNAs [150]. 

In contrast to the complete EMT process occurring during embryogenesis, EMT often halts at an intermediate state, known as partial EMT. Such cancer cells are hybrids exhibiting both epithelial and mesenchymal characteristics and markers. The expression of E-cadherin and/or N-cadherin favours cell clustering and a process known as collective cell migration [151,152]. Does E-cadherin expression also trigger ferroptosis resistance in such cell clusters? Additionally, what is the role of N-cadherin, or how is the signal integrated in clustered cells expressing both E- and N-cadherin? Finally, many tumours re-express E-cadherin in metastases, and in some tumour entities such as glioblastoma, breast carcinoma, or colon carcinoma, expression of E-cadherin is correlated with poor prognosis [153,154,155,156]. Are these tumours also resistant to ferroptosis?

Another question addresses the broad spectrum of EMT transcription factors leading to a multitude of different intracellular responses. For instance, YAP may not only promote ferroptosis by regulating ACSL4 and TfR1, but functional interaction of YAP with the transcription factor FOXO1 may induce expression of the antioxidative enzymes catalase and manganese superoxide dismutase. Conversely, activation of the Hippo pathway increases the oxidative stress response [157]. Can this discrepancy be explained by cell type specificity or by which mechanisms is the cellular response determined? Moreover, how is the crosstalk with other signalling pathways integrated in the ferroptosis network? As stated above, TAZ leads to augmented transcription of NOX4 via EMP1-p38, thereby increasing ferroptosis, and it is known that EMP1 also activates the PI3K/AKT/mTOR pathway [158,159]. 

In summary, the ferroptosis field is a fast-growing and fascinating research area, where numerous important links concerning its regulation by cell–cell contacts are expected in the near future. Last but not least, it will be interesting to await whether these molecular insights will contribute to therapeutically exploited ferroptosis in cancer treatment.
